# Antimicrobial resistance pattern: a report of microbiological cultures at a tertiary hospital in Tanzania

**DOI:** 10.1186/s12879-016-2082-1

**Published:** 2016-12-13

**Authors:** Nyambura Moremi, Heike Claus, Stephen E. Mshana

**Affiliations:** 1 Department of Microbiology and Immunology, Catholic University of Health and Allied Sciences, Mwanza, Tanzania; 2 Institute of Hygiene and Microbiology, Wuerzburg University, Wuerzburg, Germany

**Keywords:** Antimicrobial resistance, Cephalosporin-resistant gram-negative bacteria, MRSA, Tanzania

## Abstract

**Background:**

Antimicrobial resistance has been declared by the World Health Organization as a threat to the public health. The aim of this study was to analyze antimicrobial resistance patterns of the common pathogens occurring at the Bugando Medical Centre (BMC), Mwanza, Tanzania to provide data for antimicrobial stewardship programmes.

**Methods:**

A total of 3330 microbiological culture results scripts representing non-repetitive specimens reported between June 2013 and May 2015 were retrieved and analyzed for pathogens and their susceptibility patterns using STATA-11 software.

**Results:**

Out of 3330 specimens, 439 (13.2%) had positive culture. *Staphylococcus aureus* (*n* = 100; 22.8%), *Klebsiella pneumoniae* (*n* = 65; 14.8%) and *Escherichia coli* (*n* = 41; 9.3%) were the most frequently isolated bacteria. Of 78 *Staphylococcus aureus* tested, 27 (34.6%) were found to be methicillin resistant *Staphylococcus aureus* (MRSA). Rates of resistance of *Klebsiella pneumoniae* and *Escherichia coli* isolates to third generation cephalosporins were 38.5% (25/65) and 29.3% (12/41) respectively. *Staphylococcus aureus* and *Klesbiella pneumoniae* were commonly isolated from bloodstream infections while *Escherichia coli* and *Pseudomonas aeruginosa* were the predominant isolates from urinary tract and wounds infections respectively. Of 23 *Salmonella* species isolated, 22 (95%) were recovered from the blood. Nine of the 23 *Salmonella* species isolates (39%) were found to be resistant to third generation cephalosporins. The resistance rate of gram-negative bacteria to third generation cephalosporins increased from 26.5% in 2014 to 57.9% in 2015 (*p* = 0.004) while the rate of MRSA decreased from 41.2% in 2013 to 9.5% in 2015 (*p* = 0.016). Multidrug-resistant gram-negative isolates were commonly isolated from Intensive Care Units and it was noted that, the majority of invasive infections were due to gram-negative bacteria.

**Conclusion:**

There is an increase in proportion of gram-negative isolates resistant to third generation cephalosporins. The diversity of potential pathogens resistant to commonly prescribed antibiotics underscores the importance of sustained and standardized antimicrobial resistance surveillance and antibiotic stewardship programmes in developing countries.

## Background

Antimicrobial resistance (AMR) has reached a critical point and is now becoming a global threat to the public health worldwide. The World Health Organization (WHO) has declared AMR a public health threat and has urged different countries to develop action plan to combat the problem [1]. Knowledge on the epidemiology of healthcare-associated infections (HCAIs) and the antimicrobial susceptibility patterns of the isolates are crucial towards guiding empirical treatment [2]. The spectra of bacteria causing infections and their susceptibility pattern have been found to vary from one setting to another; a fact which highlights the importance of having local surveillance data for planning and implementing infection prevention and control (IPC) measures [3–5]. High income countries including the United States, have managed to decrease the burden of HCAIs by 30% through effective implementation surveillance systems [6]. A study which was conducted in a teaching hospital in Nigeria, showed the reduction of HCAIs rates from 5.8 to 2.8% in 2003 and 2006 respectively through the implementation of an effective infection control program [7]. In fighting emerging infections, surveillance data regarding AMR in Europe have been made available starting at regional to international levels via databases to ease communication between scientists [2, 8]. In Africa there are limited surveillance data irrespective of an upsurge of HCAIs and AMR clones circulating both in hospitals and communities [9–11]. At the BMC hospital in Tanzania the rate of extended spectrum beta-lactamases (ESBL) producing *Escherichia coli* increased from 25 to 50%, and that of MRSA increased from 16 to 44% between 2009 and 2014 [12–14]. Preliminary findings show significant variation of the epidemiology and evolution of these strains as evidenced by multiple clones of *Escherichia coli* carrying *bla*_CTX-M-15_ [15], causing sepsis among neonates and a new ST1797 MRSA causing wound infections [16]. Infections due to multidrug-resistant isolates have been found to be associated with increased morbidity and mortality [17, 18]. The emerging of these multidrug-resistant clones coupled with the introduction of expensive and last resort antibiotics such as carbapenems and vancomycin pose a great challenge in combating AMR at BMC and in developing countries at large. This analysis was done to assess the pattern of bacterial isolates and their susceptibility patterns from various specimens received at BMC microbiological laboratory between June 2013 and May 2015. These data are crucial in rationalizing empirical treatment and set measures for IPC, surveillance and policy change in Tanzania and in other developing countries.

## Methods

Between June 2013 and May 2015 a retrospective analysis of BMC microbiological culture results was done. BMC is a tertiary, consultant and teaching hospital located in Northwestern Tanzania. It serves a population of about 13 million people from eight regions. A total of 3387 laboratory culture results were retrieved during the observational period. Out of 3387 laboratory results, 87 scripts were excluded from the analysis due to incomplete filling of the microbiological laboratory form. Missing particulars included age, clinical history, antibiotic use, specimen type and ward number.

### Microbiological analysis

BMC clinical microbiology laboratory is accredited according to ISO 15189 by Southern African Development Community Accreditation Service (SADCAS) accreditation body with registration number MD 002. It participates in bacteriology external quality assurance coordinated by a reference laboratory in South Africa (WHO/NICD).

Clinicians were responsible for decisions regarding taking specimens for microbiological culture based on the clinical assessment of the patients. Specimens were processed following the BMC microbiology laboratory standard operating procedures (SOPs). Contaminants were defined as isolated bacteria that were more likely to be normal flora depending on the type and site where the specimen was taken. Identification of bacteria was carried out by conventional biochemical methods [19]. In case of ambiguity, analytical profile index (API) 20E or 20NE (bioMérieux, France) was used to confirm identification. Antimicrobial susceptibility testing was performed using the disk-diffusion method and interpreted according to the Clinical Laboratory Standards Institute guidelines (CLSI) [20]. Gram-negative bacteria resistant to either ceftriaxone (30 μg) or cefotaxime (30 μg) or both were termed as resistant to third generation cephalosporins. Intermediate susceptibility results were regarded as resistant in the analysis. MRSA was detected using cefoxitin (30 μg) disk and the isolate with zone of inhibition of ≤ 21 mm was confirmed phenotypically to be MRSA.

### Quality control

During the observation period, *Staphylococcus aureus* ATCC 25923, *Escherichia coli* ATCC 25922 and *Klebsiella pneumoniae* ATCC 13883 were used as quality control strains. In addition, in-house molecular typed MRSA strain and *Escherichia coli* ATCC 35218 were also used as positive controls for MRSA and third generation cephalosporin resistant organisms respectively.

### Data analysis

In the analysis of these data, children were defined as ≤ 12 years old and adults > 12 years old according to BMC admission criteria. Types of infections analyzed included; blood stream (sepsis, bacteremia), pneumonia, urinary tract, upper respiratory tract, surgical site, skin and soft tissue (abscess, pyoderma, pyomyositis, ulcer and wound), gastro-intestinal, central nervous system and cardiovascular system [21].

All demographic and clinical data as indicated in BMC microbiological request/report form were extracted and entered in Excel spreadsheet. Data were analyzed using STATA version 11 (STATA Corp LP, USA). Categorical variables were summarized as proportions and were analyzed using the Pearson’s Chi-Square test or Fisher’s exact to test statistical differences among the various groups. Odds ratios with their respective 95% confidence interval (CI) were calculated to measure the strength of associations. The *p*-value of less than 0.05 was considered statistically significant.

## Results

### Type of specimens

During the study period (June 2013 to May 2015), a total of 3330 non-repetitive specimens were analyzed; blood 2238 (67.2%), urine 307 (9.2%), cerebrospinal fluid 303 (9.1%), pus swabs 206 (6.2%), aspirates 148 (4.4%), sputum 86 (2.6%) and stool 42 (1.3%). Of the analyzed specimens, 971 (29.2%), 1888 (56.7%) and 471 (14.1%) were from year 2013, 2014 and 2015 respectively. The paediatric population contributed 67% (651/971), 47.9% (904/1888) and 74.7% (352/471) of the total specimens analyzed in 2013, 2014 and 2015 respectively.

### Culture results

Out of 3330 specimens processed during the observation period, 439 (13.2%) were found to be culture positive and 140 (4.2%) had contamination. Contamination rates were significantly more in pediatric wards than in other wards (106/1907; 5.6% vs. 34/1393; 2.4%, *p* < 0.001). Positive culture rates were 17.8% (173/971), 11.8% (223/1888) and 9.1% (43/471) in 2013, 2014 and 2015 respectively.

### Antibiotic use before specimen collection

Out of 3330 microbiological request/report forms, 1257 (37.8%) reported the patients to have had an exposure to antibiotics before the specimens were taken. Types of antibiotics documented were penicillins (22.2%, *n* = 279), gentamicin (16.15%, *n* = 203), ceftriaxone or cefotaxime (16.1%, *n* = 202), meropenem (0.9%, *n* = 12), ciprofloxacin (2.2%, *n* = 28) and unspecified (58.6%, *n* = 736).

### Pathogens and their susceptibility pattern

Out of 439 positive cultures, most frequently isolated pathogens were *Staphylococcus aureus* (22.8%, *n* = 100), *Klebsiella pneumoniae* (14.8%, *n* = 65) and *Escherichia coli* (9.3%, *n* = 41) Table 1. Majority of *Staphylococcus aureus* (61%, *n* = 61) and *Klebsiella pneumoniae* (70.8%, *n* = 46) isolates were from paediatric population (Paediatric, PREM/NICU and PICU) while the majority of *Escherichia coli* isolates (68.3%, *n* = 28) were from adults (Table 2). *Staphylococcus aureus* and *Klebsiella pneumoniae* were the most frequent isolates recovered from blood (3.6%, *n* = 80/2238 and 1.9%, *n* = 42/2238, Table 1). *Escherichia coli* (7.2%; *n* = 22/307) dominated the urinary tract infections.

**Table 1:** Bacterial isolates distribution by specimens

Pathogen	CSF	Blood	Urine	Aspirates	Pus swabs	Sputum	Stool	Total
	*n* = 303 (%)	*n* = 2238(%)	*n* = 307(%)	*n* = 148 (%)	*n* = 206(%)	*n* = 86(%)	*n* = 42(%)	*n* = 3330(%)
*Candida* species.	0 (0)	0 (0)	1 (0.33)	0 (0)	12 (5.8)	0 (0)	0 (0)	13
*Citrobacter* species.	0 (0)	11 (0.5)	4 (1.3)	0 (0)	9 (4.4)	3 (3.48)	0 (0)	27
*Escherichia coli*	1 (0.33)	9 (0.4)	22 (7.2)	0 (0)	8 (3.9)	1 (1.16)	0 (0)	41
*Klebsiella pneumoniae*	1 (0.33)	42 (1.9)	15 (4.9)	0 (0)	3 (1.45)	4 (4.65)	0 (0)	65
Other gram-positive^a^	0 (0)	4 (0.2)	3 (0.9)	0 (0)	1 (0.5)	0 (0)	0 (0)	8
Other gram-negative^b^	1 (0.33)	54 (2.4)	35 (11.4)	1 (0.67)	24 (11.65)	14 (16.3)	3 (7.14)	132
*Pseudomonas aeruginosa*	0 (0)	5 (0.22)	2 (0.7)	0 (0)	14 (6.8)	3 (3.48)	0 (0)	24
*Staphylococcus aureus*	0 (0)	80 (3.6)	1 (0.33)	0 (0)	18 (8.7)	1 (1.16)	0 (0)	100
*Salmonella* species.	0 (0)	22 (0.9)	0 (0)	0 (0)	1 (0.48)	0 (0)	0 (0)	23
*Streptococcus* species.	1 (0.33)	4 (0.2)	1 (0.33)	0 (0)	0 (0)	0 (0)	0 (0)	6
Total	4 (1.32)	231 (10.32)	84 (27.39)	1 (0.67)	90 (43.68)	26 (30.23)	3 (7.14)	439 (13.2)

^a^Other gram-positive: *Enterococcus* species. *n* = 7 and *Staphylococcus saprohyticus n* = 1

^b^Other gram-negative: Unidentified *n* = 106, *Enterobacter* species. *n* = 12, *Proteus mirabilis n* = 6, *Proteus vulgaris n* = 4, *Shigella* species. *n* = 4

*CSF* cerebrospinal fluid

**Table 2:** Bacterial isolates distribution by wards/units

Pathogen	Surgery	Paediatric	Medical	EMD	AICU	PREM/NICU	PICU	Total
	*n* = 425 (%)	*n* = 1403 (%)	*n* = 790 (%)	*n* = 95 (%)	*n* = 113 (%)	*n* = 425 (%)	*n* = 79 (%)	*n* = 3330
*Candida* species.	9 (2.1)	0 (0)	0 (0)	3 (3.2)	0 (0)	0 (0)	1 (1.27)	13
*Citrobacter* species.	10 (2.4)	3 (0.2)	7 (0.9)	0 (0)	1 (0.9)	5 (1.2)	1 (1.27)	27
*Escherichia coli*	9 (2.1)	11 (0.7)	13 (1.7)	5 (5.3)	1 (0.9)	2 (0.5)	0 (0)	41
*Klebsiella pneumoniae*	5 (1.2)	18 (1.3)	12 (1.5)	2 (2.1)	0 (0)	28 (6.6)	0 (0)	65
Other gram-positive^a^	0 (0)	3 (0.2)	0 (0)	3 (3.2)	0 (0)	1 (0.2)	1 (1.27)	8
Other gram-negative^b^	33 (7.8)	31 (2.2)	25 (3.2)	6 (6.3)	6 (5.3)	29 (6.8)	2 (2.5)	132
*Pseudomonas aeruginosa*	7 (1.7)	4 (0.3)	7 (0.9)	1 (1.1)	1 (0.9)	4 (0.9)	0 (0)	24
*Staphylococcus aureus*	17 (4)	36 (2.6)	16 (2.0)	2 (2.1)	4 (3.5)	24 (5.7)	1 (1.27)	100
*Salmonella* species.	1 (0.2)	17 (1.2)	2 (0.3)	0 (0)	1 (0.9)	1 (0.2)	1 (1.27)	23
*Streptococcus* species.	0 (0)	3 (0.2)	0 (0)	1 (1.1)	0 (0)	2 (0.5)	0 (0)	6
Total	91 (21.5)	126 (8.9)	82 (10.5)	23 (24.4)	14 (12.4)	96 (22.6)	7 (8.9)	439

*PREM* premature unit, *AICU* adult ICU, *NICU* neonatal ICU, *PICU* paediatric ICU, *EMD* emergency medicine department

^a^Other gram-positive: *Enterococcus* species. *n* = 7 and *Staphylococcus saprohyticus n* = 1

^b^Other gram-negative: Unidentified *n* = 106, *Enterobacter* species. *n* = 12, *Proteus mirabilis n* = 6, *Proteus vulgaris n* = 4, *Shigella* species. *n* = 4

Of 41 *Escherichia coli* isolates, 19 were tested for the susceptibility to third generation cephalosporins of which, 12 (63.2%) were found to be resistant. Out of 22 *Escherichia coli* isolates from urinary tract infections, 20 (90.9%) were susceptible to nitrofurantoin. Twenty four *Escherichia coli* isolates were tested for the susceptibility to ciprofloxacin; 16 (66.7%) were found to be resistant. Regarding 65 *Klebsiella pneumoniae* isolated, 31 were tested for the susceptibility to third generation cephalosporins of which, 25 (80.6%) were found to be resistant whereas of 61 *Klebsiella pneumoniae* isolates tested for gentamicin, 51 (83.6%) were found to be resistant. All 48 *Klebsiella pneumoniae* isolates tested for the susceptibility to imipenem were found to be susceptible.

The proportion of *Salmonella* species among positive cultures was 5.2% (*n* = 23/439) of which 95.7% were from blood (*n* = 22), Table 1. The majority of *Salmonella* species (82.6%, *n* = 19) were isolated from paediatric wards (Table 2). Out of 23 *Salmonella* species, 16 were tested for the susceptibility to third generation cephalosporins of which, 9(56.3%) were found to be resistant. All 15 *Salmonella* species tested against ciprofloxacin were found to be susceptible.

Most of the *Pseudomonas aeruginosa* isolates (58.3%, *n* = 14/24) were recovered from wound swabs. Of 24 *Pseudomonas aeruginosa* isolates, 16 were tested for the susceptibility to the third generation cephalosporins of which, 14(87.5%) were found to be resistant. A total of 20/22 (90.9%) *Pseudomonas aeruginosa* were found to be susceptible to amikacin.

### Methicillin-resistant *Staphylococcus aureus* (MRSA) trend

**Fig. 1:**
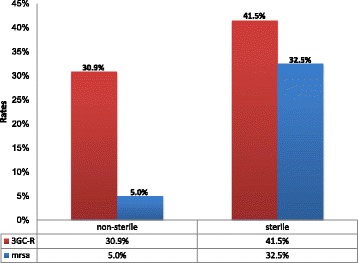
Third generation cephalosporins-resistant (3GC-R) gram negative bacteria and MRSA isolation from non-sterile Vs sterile body sites

Out of 100 *Staphylococcus aureus* isolates, 78 isolates were tested for cefoxitin of which, 27 (34.6%) were found to be MRSA. Of 27 MRSA isolates, 62.9% (*n* = 17) were from the paediatric population. The MRSA proportion kept on fluctuating during the observation period; it was 41.2% (*n* = 14/34), 47.8% (*n* = 11/23) and 9.5% (*n* = 2/21) in 2013, 2014 and 2015 respectively (*p* = 0.016). MRSA isolates were significantly more isolated from sterile parts of the body (blood, aspirates and cerebrospinal fluid) than from non-sterile (urine, pus and wound swabs) (32.5 vs. 5%; *p* = 0.01) (Fig. 1).

### Resistance to third generation cephalosporins

The overall proportion of gram-negative bacteria resistant to third generation cephalosporins was 35.9% (112/312). The majority (59.8%, *n* = 67/112) of these isolates were from children. When distributed by years of the observation, the isolation trend was 44.9% (*n* = 57/127), 26.5% (*n* = 44/166) and 57.9% (*n* = 11/19) in 2013, 2014 and 2015 respectively. The gram-negative bacteria resistant to third generation cephalosporins proportion was significantly higher in isolates from Intensive Care Units (ICUs) (46.9%) than in other wards (31.8%); OR = 1.89, 95% CI (1.1–3.3), *p* = 0.01. In addition, the isolation rate of the gram-negative bacteria resistant to third generation cephalosporins was higher from sterile sites (41.5%) than non-sterile sites (30.9%), *p* = 0.051 (Fig. 1).

## Discussion

In the present analysis, a total of 3387 specimens that were sent for culture and sensitivity between June 2013 and May 2015, the number seem to be little in a hospital with a bed capacity of 900. Relying on the National or local treatment guidelines which emphasize more on empirical treatment might have contributed to low number of specimens received in the laboratory [22]. Deployment and adherence to antimicrobial therapy guidelines and policies using evidence-based generated data might improve the current situation as previously shown in Botswana [23]. Blood specimens contributed to more than half of specimens processed and the majority were from paediatric wards. This could be explained by the local practice in these wards that emphasize on the need of blood culture to all children presenting with fever.

In the present analysis, *Staphylococcus aureus* and *Klebsiella pneumoniae* were the predominant organisms causing bloodstream infections from pediatric wards. These findings are in agreement with a prospective study which was conducted at a national hospital in Tanzania among neonates with fever which found *Staphylococcus aureus* to be the predominant pathogen (54.5%) followed by *Klesbiella pneumoniae* (32.7%) [24]. Similar findings were observed at BMC in 2010 [12], indicating the persistence of pattern in pediatric wards especially in neonatal units.

With regard to urinary tract infections in this analysis, *Escherichia coli* was found to be the most frequently isolated pathogen. A similar finding was observed in previous studies done in the same setting [25, 26]. *Escherichia coli* isolates from urine demonstrated high rates of resistance to ciprofloxacin and third generation cephalosporins than to nitrofurantoin. The rate of resistance of nitrofurantoin has remained low all years in the present study. Despite being cheap, this antibiotic is not commonly self-prescribed. Ciprofloxacin and third generation cephalosporins are commonly used at BMC than nitrofurantoin and they are also commonly self-prescribed in the community. The overuse of ciprofloxacin and cephalosporins might be the most probable cause of higher resistance rates observed.

As in previous study [27], the majority of *Salmonella* species isolates were isolated from blood in this study and all of the tested *Salmonella* species isolates were susceptible to ciprofloxacin. This finding is similar to a study done in Ghana, which observed *Salmonella typhi* to be the most prevalent pathogen isolated from bloodstream infection and all isolates were susceptible to ciprofloxacin [27]. The observation of 100% susceptibility of *Salmonella* species isolates to ciprofloxacin in our setting could be explained by the non-use of this antimicrobial agent in paediatric wards. This can be evidenced by the fact that the majority of these isolates were from the paediatric wards. This is further supported by the previous study which observed 8% of *Salmonella* species isolates from adults to be resistant to ciprofloxacin [28]. In this study, a significant proportion of *Salmonella* species isolates were resistant to third generation cephalosporins. Similar findings have been observed in Europe from people who travelled from Asian countries [29, 30] and in Kenya [31]. However, these findings are in contrast with the previous study conducted three years ago in the same hospital which observed all *Salmonella* species to be susceptible to ceftriaxone [28]; suggesting the presence of emerging multidrug-resistant *Salmonella* species in Northwestern Tanzania.

As in previous study [14], most of the *Pseudomonas aeruginosa* isolates in this study were recovered from wound swabs and more than 50% were resistant to third generation cephalosporins. The majority of *Pseudomonas aeruginosa* isolates in this study were sensitive to amikacin, as previously observed [32]. This could be explained by the rare use this aminoglycoside in our setting and other setting in low and middle-income countries.

The general proportion of MRSA in this study was found to be in agreement with MRSA proportion observed in other studies done in the same setting in 2010 and 2014 [12, 14]. However, during the observation period a significant decrease was observed in 2015, this could be explained by the ongoing hand washing campaigns and improvement of IPC programs in various BMC wards coupled by increased awareness of the emergence of multidrug-resistant strains among healthcare workers. The latter measures have been shown to reduce MRSA outbreaks [33]. In the present study, MRSA isolates were significantly found to cause invasive infections. Invasive MRSA infections might lead to increased morbidity and mortality of the infected patients as previously observed [12].

Although the overall trend of the proportions of gram-negative bacteria resistant to third generation cephalosporins appeared to decrease in 2014 in comparison to 2013, the significant upsurge of these notorious strains was observed in 2015. The high resistance rates observed in ICU patients could be explained by the fact that patients in ICU tend to stay longer in the hospital and this has been found to increase risk infections due to multidrug-resistant pathogens [34].

## Conclusions

The observed high proportion of gram-negative isolates being resistant to third generation cephalosporins is alarming and calls for a surveillance of HCAIs and community infections to establish the source and transmission pathways. The emergence of *Salmonella* species isolates resistant to third generation cephalosporins is worrisome because of increased infections of *Salmonella* species observed in HIV infected patients which is endemic in low-income countries.

Despite the good service offered by BMC microbiology laboratory shortcomings have been observed which need rectifications. Monitoring and proper screening of isolates resistant to third generation cephalosporins to know the mechanisms of resistance responsible should be done. Consistency in antimicrobials tested for the isolates should be observed to establish a homogeneous database. Adherence to CLSI guidelines in processing microbiological isolates should be mandatory. It is a high time to consider having the local antimicrobial guide using local generated data to guide treatment. There is need to perform molecular characterization of common isolates involved in HCAIs in developing countries so that evidence based data can be used to improve the IPC in our setting.

### Study limitations

The analysis based on getting information from microbiological request/report forms therefore it was difficult to tell whether the infection originated from the community or it was healthcare-associated. Antimicrobial susceptibility testing depended on the availability of antimicrobial discs at that particular time. This has resulted to inconsistent bacterial antibiogram patterns and difficulties in reporting resistance rates of the most frequent isolates. Antimicrobial agents were tested against wrong isolates as per CLSI guidelines e.g. ceftriaxone was tested against *Pseudomonas aeruginosa* isolates. Moreover, specific tests like inducible clindamycin resistance (D-test) and ESBL testing were not done. Other important epidemiological information such as patient’s outcome, duration of hospital stay etc. were not reported. Lastly, there might be sampling bias in this analysis because there were no guidelines for systematic specimen collection for microbiological culture.

## Abbreviations

AMR: Antimicrobial resistance
API: Analytical profile index
BMC: Bugando Medical Centre
CLSI: Clinical laboratory standards institute
ESBL: Extended spectrum beta lactamase
HCAIs: Healthcare-associated infections
IPC: Infection prevention and control
MRSA: Methicillin-resistant *Staphylococcus aureus*
NICD: National institute for communicable diseases, a division of the national health laboratory service, South Africa
NICU: Neonatal intensive care unit
PICU: Paediatric intensive care unit
PREM: Premature unit
SADCAS: Southern African development community accreditation service
WHO: World Health Organization
